# Extracranial Sources of S100B Do Not Affect Serum Levels

**DOI:** 10.1371/journal.pone.0012691

**Published:** 2010-09-10

**Authors:** Nancy Pham, Vincent Fazio, Luca Cucullo, Qingshan Teng, Peter Biberthaler, Jeffrey J. Bazarian, Damir Janigro

**Affiliations:** 1 Cleveland Clinic Lerner College of Medicine of Case Western Reserve University, Cleveland Clinic Foundation, Cleveland, Ohio, United States of America; 2 Department of Cell Biology, Cleveland Clinic Foundation, Cleveland, Ohio, United States of America; 3 Neurological Surgery, Cleveland Clinic Foundation, Cleveland, Ohio, United States of America; 4 Surgical Department Munich Central, Ludwig-Maximilians-Universität Munich, Munich, Germany; 5 Department of Emergency Medicine, University of Rochester School of Medicine, Rochester, New York, United States of America; Hungarian Academy of Sciences, Hungary

## Abstract

S100B, established as prevalent protein of the central nervous system, is a peripheral biomarker for blood-brain barrier disruption and often also a marker of brain injury. However, reports of extracranial sources of S100B, especially from adipose tissue, may confound its interpretation in the clinical setting. The objective of this study was to characterize the tissue specificity of S100B and assess how extracranial sources of S100B affect serum levels. The extracranial sources of S100B were determined by analyzing nine different types of human tissues by ELISA and Western blot. In addition, brain and adipose tissue were further analyzed by mass spectrometry. A study of 200 subjects was undertaken to determine the relationship between body mass index (BMI) and S100B serum levels. We also measured the levels of S100B homo- and heterodimers in serum quantitatively after blood-brain barrier disruption. Analysis of human tissues by ELISA and Western blot revealed variable levels of S100B expression. By ELISA, brain tissue expressed the highest S100B levels. Similarly, Western blot measurements revealed that brain tissue expressed high levels of S100B but comparable levels were found in skeletal muscle. Mass spectrometry of brain and adipose tissue confirmed the presence of S100B but also revealed the presence of S100A1. The analysis of 200 subjects revealed no statistically significant relationship between BMI and S100B levels. The main species of S100B released from the brain was the B-B homodimer. Our results show that extracranial sources of S100B do not affect serum levels. Thus, the diagnostic value of S100B and its negative predictive value in neurological diseases in intact subjects (without traumatic brain or bodily injury from accident or surgery) are not compromised in the clinical setting.

## Introduction

S100B, a protein produced primarily by brain astrocytes, is an established peripheral biomarker of blood-brain-barrier permeability associated with various CNS (central nervous system) injuries [1], [2]. Elevated levels accurately reflect the presence of neuropathological conditions including traumatic head injuries [3]–[5], psychiatric disorders [6], cerebrovascular insults [7] and neurodegenerative diseases [8], while normal levels reliably exclude major CNS pathology [4], [9], [10]. Its potential clinical use in the therapeutic decision making process is substantiated by a vast body of literature validating variations in serum 100B levels with standard modalities for prognosticating the extent of CNS damage: alterations in neuroimaging, cerebrospinal pressure, and other brain molecular markers (neuron specific enolase, glial fibrillary acidic protein) [11], [12]. Thus, the major advantage of using S100B is that elevations in serum can be easily measured, providing a sensitive measure to help rule out major CNS dysfunction.

An important application of serum S100B testing is the selection of patients with minor head injury who do not need further neuroradiological evaluation, as studies comparing CT (computerized tomography) scans and S100B levels have demonstrated S100B values below 0.1 ng/mL are associated with low risk of obvious neuroradiological changes (such as intracranial hemorrhage) or significant clinical sequelae [10]. In addition, several papers have shown that the high negative predictive value (NPV = TN/(TN+FN), where TN =  true negative and FN =  false negative) of S100B in several neurological conditions is due to the fact that serum S100B levels reflect blood-brain barrier permeability changes even in absence of neuronal injury [1]–[9]. Thus, serum S100B levels may increase prior to a significant change in neurological function, or neuronal cell death, which is in accordance to its function as an astroglial and non-neuronal protein marker. This is an important clinical finding inasmuch S100B levels in the normal range rule out cerebrovascular damage and injury to the CNS in nearly 99% of patients by neurological imaging [10]–[13].

Recently, controversy has arisen with regards to the brain specificity of this protein. Several lines of evidence suggest that extracerebral sources contribute to S100B serum levels [13]. For example, in multi-organ system trauma without traumatic head injury, elevations in serum S100B levels collectively reflect traumatized fat, muscles, or bones since the blood-brain barrier remains intact in these situations and therefore, cannot account for the systemic rise in S100B levels. Similarly, shed blood from cardiac surgery displayed heightened levels without apparent head injury [14]. Marathon runners, joggers, basketball players, ice hockey players, and boxers also have high S100B levels after engaging in their respective physical activity, which may be the result of both BBB (blood-brain barrier) opening and muscular release [15]–[17].

The objective of this current study was to determine if tissue sources, outside the brain, contribute significantly to serum levels of S100B. In this study, we characterized by Western blot the expression of S100B in human tissue using two different antibodies and corroborated this data with mass spectrometry. We then assessed how expression in these tissues affects the current clinical methods for detection of S100 by using the LIAISON® S100 or LIAMAT systems (Sangtec-Diasorin), and the Elecsys system (Roche Diagnostics). Finally, we analyzed 200 subjects to determine if adiposity or fat S100B affected serum levels.

## Methods

### Human Subjects

All studies were performed in accordance with the Declaration of Helsinki and written approval by the IRB Committee at the Cleveland Clinic. All samples (blood and tissue) were obtained by written informed consent and given a coded identifier to retain subject anonymity. Tissue samples were obtained as surplus tissue from various medical procedures conducted at the Cleveland Clinic, generously provided by the department of Pathology. For the purpose of this study we analyzed 200 subjects, consisting of 155 patients and 45 controls (Table 1). Note that our patient population comprised of individuals with a broad spectrum of ages, races, and pathologies, which ranges from psychiatric disorders to brain tumors. We also recruited a large number of volunteers without any pathology or psychiatric condition, as detected by a psychiatrist delivering a brief psychiatric exam. Samples were analyzed by different techniques, the details of which are presented in the Methods Section and below. We analyzed tissue protein contents by antibody-dependent assays or by mass spectrometry (MS). When possible, two antibodies were used for the detection of different antigen regions in the S100B protein. BMI (body mass index) was calculated based on the weight (in kilograms) divided by the square of the patient's height (in meters) taken from the medical record.

**Table 1:** Patient Characteristics.

Grouping	N	Age	Age Range	Height (cm)	Height Range (cm)	Weight (kg)	Weight Range (kg)	BMI (kg/m^2^)	BMI Range (kg/m^2^)	S100B (ng/mL)	S100B Range (ng/mL)	% Female	Caucasian	African American	Other
Ped. Controls	20	16.6(1.1)	14–18	171.3(7.3)	161–191	67.3(16.9)	45–118	22.9(5.1)	16.7–38.4	**0.11(0.09)**	**0.03**–**0.42**	55.0	14	1	5
Ped. (Non-Psych)	25	14.3(3.3)	8–18	156.9(18.9)	120–185	65.6(28.9)	21–111	25.3(7.7)	12.9–38.5	**0.23(0.17)**	**0.05**–**0.70**	40.0	19	1	5
Ped. Psychotic	39	14.3(2.6)	10–18	162.6(13.4)	137–186	68.3(29.0)	27–205	25.4(9.5)	11.3–76.2	**0.26(0.23)**	**0.02**–**1.11**	43.6	18	16	5
Adult Controls	25	39.1(11.1)	22–60	169.1(8.1)	147–183	68.1(9.3)	50–82	23.8(2.8)	19.1–31.8	**0.08(0.05)**	**0.04**–**0.27**	40.0	13	1	11
Adult (Non-SVID)	22	62.5(9.5)	47–80	174.1(10.1)	155–189	79.1(18.4)	54–127	26.1(6.1)	19.3–47.8	**0.22(0.29)**	**0.01**–**0.13**	27.3	20	2	0
Adult (SVID)	59	65.8(8.0)	49–85	169.6(10.5)	146–203	78.8(16.7)	37–125	27.3(5.1)	15.6–40.7	**0.18(0.15)**	**0.01**–**0.71**	40.7	53	6	0
BBBD	10	53.6(15.8)	21–72	165.3(7.8)	152–178	73.8(23.0)	29–105	26.8(7.4)	10.6–36.0	**0.14(0.04)**	**0.07**–**0.22**	80.0	10	0	0

This table describes the patient characteristics of our study patient populations [mean value +/− (standard deviation)].

### BBB Disruption

The Cleveland Clinic Brain Tumor Institute provides a treatment called blood-brain barrier disruption for primary CNS lymphomas. All procedures were performed after informed consent was obtained using protocols approved by the Cleveland Clinic Foundation IRB. In this protocol, intra-arterial mannitol (1.4 M) is administered via a carotid or vertebral artery, and BBB disruption was confirmed by contrast CT immediately after chemotherapy. The details are described elsewhere [18].

### Tissue Protein Extraction

Proteins were extracted from various tissues using the Millipore Total Protein Extraction Kit (Chemicon subsidiary, Temecula, CA). Briefly, tissues were weighed, chopped into small pieces, and kept on dry ice. Then 1X TM buffer [13 mL of HEPES (pH 7.9), MgCl_2_, KCl, EDTA, sucrose, glycerol, sodium deoxycholate, NP-40, sodium orthovanadate] and a protease inhibitor cocktail was added to each tissues at 2.5 mL per gram of tissue and put on ice for 5 minutes. The tissue was homogenized for 20 seconds and then put on dry ice for 15 seconds. This cycle was repeated 3 times. The homogenized tissues were rotated at 4°C for 20 minutes and centrifuged at 11,000 rpm at 4°C for 20 minutes. The supernatant was collected and stored at −80°C until analyzed further.

### Western Blots

**Figure 1:**
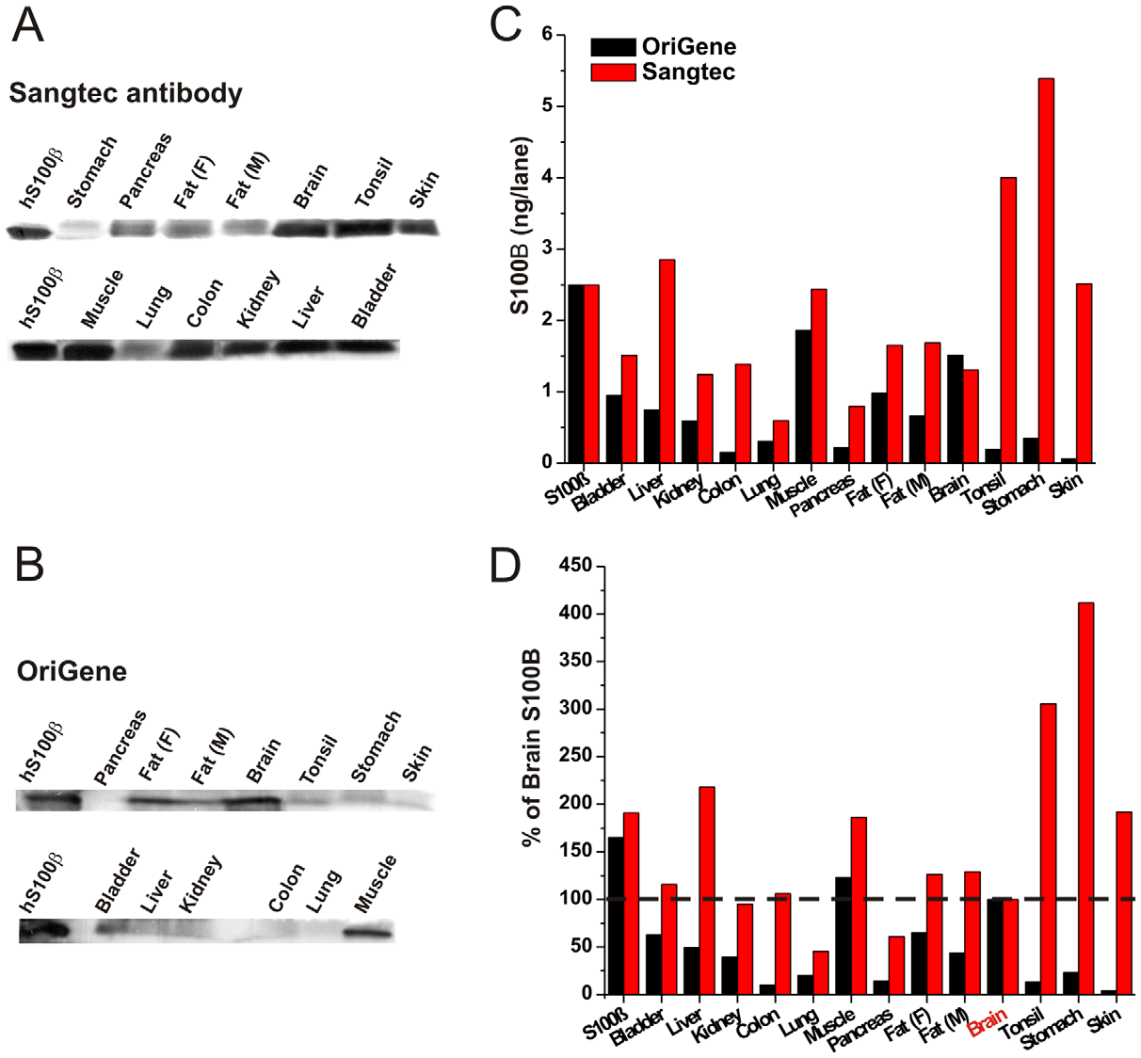
S100B Antibody Comparison. Twelve different types of human tissues were assessed for S100B expression by two different antibodies by Western blot. The OriGene monoclonal antibody was made by immunizing against a synthetic peptide corresponding to residues on the C-terminus of human S100B. The polyclonal Sangtec antibody was raised against the whole human protein. (A) shows the tissue specific expression level of S100B using the Sangtec-Diasorin antibody and (B) OriGene antibody after Western blot analysis. Regardless of the antibody used, S100B was found in tissues other than brain. (C) We quantified and compared the results of the two Western blots obtained by the two different antibodies as well as (D) this data normalized to brain tissue. The rank order of S100B expression is different depending on the antibody used.

**Figure 2:**
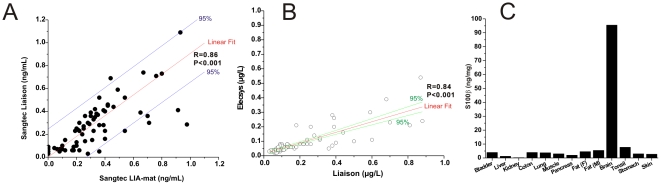
Extracranial Detection of S100B. Extracranial sources of S100B revealed on Western blots do not affect the clinical detection of S100B using the Sangtec-Diasorin immunoassay. (A) The results of two clinically relevant Sangtec Diasorin immunoassay systems; the fully automated Liaison (x-axis) was compared with the manual LIA-mat assay kit. There was good correlation between the two systems. (B) A good correlation existed between two automated (Elecsys by Roche Diagnostics and Liaison by DiaSorin) immunoassays for S100B. (C) The same human tissue protein extracts used for Western blotting previously were analyzed with the Sangtec-Diasorin immunoassay. In contrast to what we found in Western blots, the brain is the main chief expressor detected by this method.

Protein concentration was determined by the Bradford assay method (Bio-Rad, Hercules, CA). Total proteins (50 µg/lane) were separated on 10–20% polyacrylamide gels with SDS-PAGE at 80 V and transferred onto a polyvinylidene difluoride membrane (Millipore Corp., Bedford, MA) by electroblotting at 100 V of constant voltage for 1 hour. After blocking with TBST and milk (Tris-buffered saline, 0.05% milk powder, and 0.05% Tween 20) for at least 2 hours, the membrane was probed overnight at 4°C either with the Sangtec-Diasorin or OriGene S100B primary antibody (1∶1000). The OriGene monoclonal antibody was made by immunizing against a synthetic peptide corresponding to residues on the C-terminus of human S100B. The polyclonal Sangtec antibody was raised against the whole human protein. These antibodies were selected because they target different regions of the S100B protein (see legend of Figure 1). The Sangtec antibody was also chosen for Western blot analysis because it constitutes the capture antibody of the LIAISON kit (Figures 2A, B). After a series of washes, the membrane was incubated with secondary horseradish peroxidase-conjugated anti-goat IgG antibody (rabbit or rat) for 2 hours. Western blots were visualized by enhanced chemiluminescence reagent (ECL Plus, Amersham Biosciences, Piscataway, NJ). The dilution for the Sangtec antibody, which is not commercially available, was derived from a previous study [14].

### Relative Density on Western Blots

Western blots were scanned on a scanner interfaced with a PC using HP Precision Scan Pro 3.02 analysis software (Hewlett-Packard Co., Palo Alto, CA). The scanned grayscale images were saved in an uncompressed TIFF format and further analyzed using software specifically designed for measuring grayscale image density, developed by Nonlinear USA, Inc. (Durham, NC) PhoretixTM ID (Version 2003.01).

### Immunoassay Determination

We used colorimetric immunosorbent assay, Sangtec® 100 ELISA, by DiaSorin, Inc. (Stillwater, MN) to quantify S100B. The limit of detection is 0.03 ng/mL. We also used the Canag/Fujirebio system by Fujirebio Diagnostics, Inc. (Tokyo, Japan) to measure various mono- and hetero- dimers of S100B. The Elecsys system (Roche Diagnostics, Indianapolis, IN) was used as well for the purpose of measuring S100B.

### Mass Spectrometry

We used a LC-MS system Finnigan LCQ-Deca ion trap mass spectrometer system with a Protana microelectrospray ion source interfaced to a self-packed 10 cm×75 um id Phenomenex Jupiter C18 reversed-phase capillary chromatography column. Data were analyzed by using all CID spectra collected in the experiment to search the National Center of Biotechnology Information (NCBI) non-redundant database with the search program TurboSequest. All matching spectra were verified by manual interpretation. The interpretation process was also aided by using the programs Mascot and Fasta to perform additional searches, as needed.

### Statistical Methods

Data are presented as mean ± standard deviation (SD). JMP® 8.0 (Cary, NC) was used for statistical analysis. Correlation plots were produced using statistical software by the Origin Lab Corporation (versions 7.0 and higher, Northampton, MA) to calculate correlation coefficients (R) and 95% confidence limits. Significant difference or correlation was assessed by P-values of <0.05, calculated using the Student's t-statistic.

## Results

We first analyzed S100B protein content in a variety of tissue samples (Table 2). Western blot analysis revealed that, regardless of the antibody used, S100B is found in tissues other than brain. The results in Figure 1A and 1B show protein signals obtained with the Sangtec or OriGene antibody.

**Table 2:** Surgical Specimen Characteristics.

Tissue Specimen	Primary Diagnosis	Sex	Age	Race
Bladder	Bladder Cancer	M	65	Caucasian
Liver	Liver Cancer	M	57	Caucasian
Kidney	RCC	M	71	Caucasian
Colon	UC	M	53	Caucasian
Lung	Lung Cancer	M	80	Caucasian
Muscle	Sarcoma	M	67	Black
Pancreas	Cyst	F	42	Caucasian
Fat	Sarcoma	F	81	Black
Fat	Crohn's Disease	M	50	Caucasian
Brain	Epilepsy	M	35	Hispanic
Tonsil	Hypotrophy	F	33	Caucasian
Stomach	Unknown	M	54	Caucasian
Skin	Thigh Mass	M	43	Black

Table 2 details the patient characteristics of the surgical specimens used for Western blotting and ELISA analysis. RCC =  Renal Cell Carcinoma, UC =  Ulcerative Colitis.

The results presented thus far were obtained with two different antibodies directed towards the same antigen, yet these results were not quantitatively consistent. To rule out that the measured signal may be due to protein other than S100B, we analyzed the molecular identity of selected lanes shown in Figure 1A and 1B by MS.

The two antibodies gave comparable but not identical results. While both revealed a strong signal from brain tissue, muscle and fat also exhibited strong levels of expression. The data were quantified to construct the bar graph shown in Figure 1C where the values are expressed in nanograms per lane. An added standard of S100B was used to quantify S100B expression in tissues. In addition, we compared these expression levels to brain tissue as shown in Figure 1D. Note that when data are normalized to brain expression, significant levels were seen in extracranial tissues and the extent of this phenomenon depended on the antibody used.

### Molecular Identity of the S100B Signal

MS analysis confirmed that the brain tissue signal, consistent by molecular weight with S100B, was indeed this protein (Table 3). When the same lanes were isolated from fat samples, S100B expression was similarly found. We chose fat tissue because it was most consistently reported in the literature to contribute to elevated serum S100B levels [19], [20]. In addition to S100B, MS analysis also revealed the presence of another protein of the S100 family, namely S100A1 [21].

**Table 3:** Mass Spectrometry Characterization of Protein Bands.

Tissue Type	Proteins Detected	Nomenclature
Human Brain	S100B, S100A	S100 calcium binding protein A1 (Acc# 5454032,11 kDa)S100 protein, beta polypeptide (Acc# 5454034, 11 kDa)
Human Fat (F)	S100B, S100A	S100 calcium binding protein A1 (Acc# 5454032, 11 kDa)S100 protein, beta polypeptide (Acc# 5454034, 11 kDa)
Human Fat (M)	S100B, S100A	S100 calcium binding protein A1 (Acc# 5454032, 11 kDa)S100 protein, beta polypeptide (Acc# 5454034, 11 kDa)

Mass spectrometry analysis revealed that human brain and fat tissue contained S100B as well as the presence of another protein of the S100 family, namely S100A.

### Results with a Clinically Relevant Platform

Most of the clinical results dealing with the utilization of S100B as a predictor of neurological disorders were obtained with one of several immunoassays that are commercially available (for a complete recent summary see Goncalves et al., [13]).

We measured S100B by ELISA (Sangtec-LIAISON; Figure 2C). With the LIAISON system, brain was the chief expressor, while other tissues contributed less significantly, if at all to the signal. In particular, neither muscle nor fat gave a significant signal as it did in the Western blots. Thus, tissue samples S100B content was substantially different when using ELISA vs. Western (compare Figure 2C and Figure 1C).

In addition to this commercially available immunoassay, previous literature dealing with clinical samples has often used an automated version of the same test [13]. The results of a direct comparison between the automated and manual test are shown in Figure 2A. Note that a good correlation existed between the two tests. In addition, we compared the results of a clinically relevant test based on the Sangtec-Diasorin platform with another commonly employed platform (Roche Diagnostic, Figure 2B). Again, we noted a good correlation between the two systems. These results have shown that there is a significant quantitative difference when comparing results obtained by gel-based analysis versus ELISA-type immunoassay systems. The latter, produced comparable results both quantitatively and qualitatively, suggesting that the use of these clinically relevant platforms leads to meaningful and comparable results.

### Molecular Nature of S100B Released by Brain Tissue

The literature dealing with the detection of S100B as an indicator of neurological dysfunction or BBB leakage was profoundly affected by the discovery of Marchi, et. al [18] who demonstrated that upon iatrogenic BBB disruption (BBBD) S100B levels were elevated minutes after the procedure itself. This prompted us and others to characterize S100B as an indicator of BBB damage rather than a protein related to neuronal cell death or other types of brain injuries [1], [2], [22]–[30]. However, recent findings by others [1], [2], [13], [17], [19], [22]–[35] have shed doubt on the utility of this approach, primarily because of extracranial sources of S100B. Our data, in fact, show that this is indeed the case and that tissue other than brain expresses this protein. However, it is also known that S100B may be detected in its monomeric or dimeric form. In addition, S100B may form a homo- or hetero- dimer with its companion S100A1 [21], [36].

**Figure 3:**
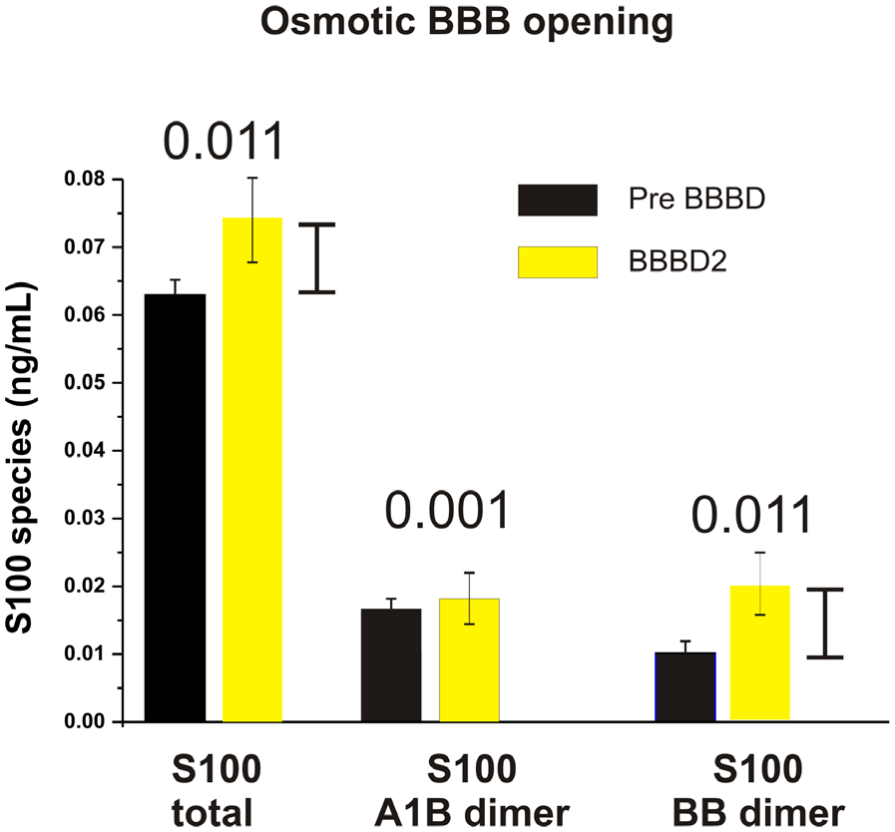
BBB Opening: Comparison of Various Forms of S100B. The main species of S100B released from BBB disruption is the B-B dimer as measured using the CanAg/Fujirebio ELISA system. The rise in total S100B after BBB opening was 0.011 ng/ml, which was found to be accounted for by the concomitant rise of the B-B dimer. There was virtually no change in the concentration of the A1-B dimer with BBB opening. We determined the concentration of the A1-B dimer by using the CanAg S100A1B EIA solid-phase, two-step, non-competitive immunoassay based on two mouse monoclonal antibodies specific for two different epitopes specifically expressed in S100A1B. The assay thus determines S100A1B with very low cross-reactivity with S100BB or other forms of S100. Similarly, to measure the B-B dimer we used the CanAg S100BB EIA solid-phase, one-step, non-competitive immunoassay based on two mouse monoclonal antibodies specific for two different epitopes specifically expressed in S100BB. This assay thus determines S100BB with very low cross-reactivity with S100A1B or other forms of S100.

We therefore wished to investigate the molecular nature of the S100B extravasating from the human brain under conditions of iatrogenic BBB disruption (Figure 3). To this end, we utilized ELISA platforms manufactured by Canag/Fujirebo to specifically dissect out the signal components due to homo- or hetero-dimers of S100B and S100A1. In Figure 3, note that BBBD caused an increase of S100B dimer but not of the S100A1-B heterodimer. The increase of the B-B homodimer was the principal event that caused the elevation of the total signal. In other words, these results demonstrated that the main species of S100B released by the brain when the blood-brain barrier is disrupted is the S100 B-B. The numbers above the histogram refer to ng/mL changes occurred between pre-BBBD and at the time when chemotherapy was injected. Note that virtually all the increase in total S100B measured was due to the B-B dimer (0.011 ng/ml). The quantitative results for total S100 are similar to what published previously [2].

### Do extracranial sources contribute to the clinical test for serum S100B?

**Figure 4:**
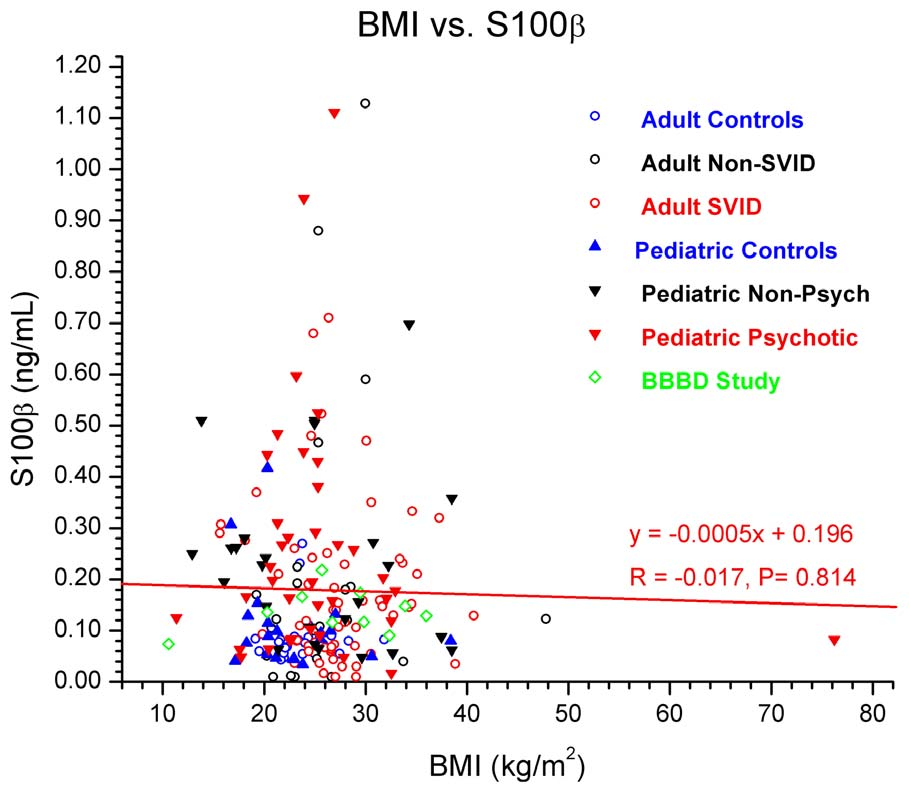
Correlation Between BMI and S100B. Given the expression of S100B in adipocytes, we investigated the relationship between fat content and S100B levels using 200 subjects. No correlation was found between BMI and S100B levels.

The results we have presented so far unveiled a complex scenario where several different molecular species act, upon BBBD, to modify serum values of S100B. We therefore wished to investigate the S100B values in serum of patients or controls across, to our knowledge, the broadest published population of subjects. The results are summarized in Figure 4 and Table 1. Given the expression of S100B in adipocytes, we investigated the relationship between fat content and S100B levels. This was achieved by determining body mass index (BMI; see Methods for calculations). Note that when serum samples from 200 subjects were analyzed, no correlation was found between BMI (or body weight in kg, not shown) and S100B levels (Figure 4). This suggests that while unquestionably S100B is present in fat, it does not alter the S100B serum concentration in “normal” volunteers, patients affected by a variety of disorders, or pediatric controls or children with various illnesses. These results are also consistent with findings by Biberthaler et al. [27], [37]–[40] who evaluated the relevance of BMI in serum values of S100B in a large number of controls and traumatic brain injury patients.

## Discussion

The main contribution of this article to our understanding of S100B use in the clinical setting is that in spite of robust expression by extracranial sources, changes in serum levels are primarily dictated by extravasation across the disrupted BBB. In addition, we have shown that the main molecular species of total S100B related to blood-brain barrier disruption is the S100B homodimer.

### Methodological considerations

To our knowledge, this is the most exhaustive study ever performed to compare and cross-validate various approaches to the detection of S100B. In addition, we have used a vast array of subjects and tissues. Western blot analysis revealed that, regardless of the antibody used, S100B is found in tissues other than brain. Our results show, surprisingly, a poor correlation between different antibodies. While cross-reactivity of antibodies is well known and widely accepted, we were nevertheless surprised that the rank order of expression depended on the antibodies used. In addition, testing by ELISA showed a different profile, where expression by extracranial sources was less prominent. We were, however, able to show that the gel-based approach was sensitive for S100B which was detected by MS. The co-expression of S100A1 was expected, based on results by others [41]. Although discussion of the possibilities of why we observed such varying expressions of S100B protein between the platforms of Western blot and ELISA is a very noteworthy, adequate analysis is beyond the scope of this paper. These findings may have implications beyond recent S100B research efforts, inasmuch as most of our current knowledge on protein function is based on antibody detection of levels by gel electrophoresis. The use of antibody-independent detection therefore appears advisable.

### Limitations and strengths for basic scientists

As stated above, our results are not comforting for the basic scientist. Western blotting is an easy and reproducible technique that enjoys wide popularity. Our concerns are not unique [42]. We found different levels of S100B expression by using two different antibodies and found poor qualitative and quantitative correlation. Similar results were obtained in rodent tissue with other commercially available antibodies (*data not shown*). Such discrepancies may pose a significant problem, as they substantially impinge on and alter the conclusions drawn from experiments including those aiming at discoveries of disease treatment modalities or with diagnostic purpose.

Another practical limitation of this study, due to the diversity and number of patients included, was the use of the BMI calculation to assess individuals' relative body fat. Recent studies on nutrition and metabolism have validated techniques such as ultrasound, air displacement plethysmography and bioelectrical impedance to be superior to BMI for accurately measuring body fat [43]. These techniques were not readily available nor could we easily implement them and therefore utilized the BMI calculation not only out of practicality, but also out of the widespread use of it in other S100B studies.

Based on these observations, it may be desirable to incorporate any or all of the following to ascertain that an observed signal is indeed reflective of S100B: 1) Protein levels are generally caused by increased mRNA. Therefore, if release of S100B is due to a pathology initiating active synthesis rather than by passive release from necrotic astroglial cells (as in stroke) quantitative RT-PCR of S100B messenger RNA would be appropriate to further support any observations; 2) Antibody-independent methods such as MS should be included for validation of target recognition; 3) When possible HPLC (high-performance liquid chromatography) should be used in parallel or serial experiments.

### Clinical significance

While the results we present may be disappointing for the bench researcher, we also demonstrated that the clinical detection of S100B is more reproducible and robust. In addition, the type of instrument, or the platform used did not alter the results, nor did it affect the predictive value of the test. The clinical tests all measure total S100B, regardless of it monomeric or dimeric state, nor do they consider whether S100B is bound to S100B or S100A1. Preliminary results with tests detecting only S100B-S100B dimers have demonstrated, as expected, that the ceiling for “normal values” is significantly lower than the published “0.1 ng/ml” dogma [1], [6], [10], [14], [19], [24], [40], [44]–[46].

We confirm that S100B was not exclusively produced by CNS cells. We found that muscle and fat were chief extracranial expressors, which is consistent with the literature [19]. However, our results are in sharp contrast with the findings linking serum S100B to BMI [20]. These results used the same system used by us (LIAMAT), but their sample size was significantly smaller. In addition, no cross-validation with other detection systems was used. Our range of BMI values was larger, the ages broader, and the racial samples were balanced to reflect the general US population. We also included diseased patients' samples, to add to the clinical significance of the findings. We found no correlation between S100B and weight or height, but found, as expected, a correlation with age [47]. When the values were restricted within a given category (*e.g.*, pediatric controls in Table 1), we still found no correlation between BMI and S100B. Why these results are in sharp contrast with Steiner et al. [20] remains at present unknown.

Our results show that extracranial sources of S100B do not significantly affect serum levels. Thus, the reported low sensitivity and positive predictive value (relative to the reported strong specificity and negative predictive value) for S100B [24] is not apparently due to extracranial release of the protein. Further studies are needed to explore the use of S100B dimers as tools in neurodiagnostics.

There are two possible explanations that may account for the discrepancy between previous studies documenting elevated S100B levels from extracranial sources and our present findings. There have been recent reports that serum S100B levels are positively correlated with body mass index without evidence of traumatic brain injuries. Interestingly, obesity has been hypothesized to be a state of heightened systemic oxidative stress and inflammatory response, which is mechanistically linked to other co-morbid conditions such as hypertension and small vessel disease. Therefore, it is not clear whether obesity itself or obesity-associated comorbidities contribute to a rise in serum S100B in the previous studies. In our study, we directly measured the tissue specific expression of S100B in addition to serum S100B, which represents a collective source from multiple disease processes. Our study showed that an increase in fat mass might not in isolation be a major contributor to elevated S100B levels. Rather, obesity-related diseases are likely contributors. For example, small vessel ischemic disease associated with obesity is a source of serum S100B [24], [48].

Prior studies have shown that cardiothoracic surgeries resulted in higher serum levels of S100B. However, Fazio et al. demonstrated that S100B antibodies from certain ELISA kits might cross-react with other proteins found in serum[14]. It is difficult to characterize biomarkers in serum because of the wide range of protein concentrations and predominance of 10 to 20 proteins (albumin, immunoglobulins, etc.) that overwhelm the less abundant signals. This may suggest that in other studies yet unknown cross-reactants were artificially increasing the apparent S100B levels measured in serum.
