# Correction: Extracranial Sources of S100B Do Not Affect Serum Levels

**DOI:** 10.1371/annotation/bdcb41f2-a320-4401-a6ab-86e71738597e

**Published:** 2010-10-29

**Authors:** Nancy Pham, Vincent Fazio, Luca Cucullo, Qingshan Teng, Peter Biberthaler, Jeffrey J. Bazarian, Damir Janigro

In our recent article [1], we did not declare several potential competing interests relevant to this work. We apologize for this omission and would like to disclose the following information:

Dr. Janigro has received royalty payments from Roche Diagnostics and holds a patent on S100B for its use as a serum marker for blood-brain barrier leakage.

The study was partly supported by Roche Diagnostics. The company does not currently develop any products related to the results described in the article.

Reference:

1. Pham N, Fazio V, Cucullo L, Teng Q, Biberthaler P, Bazarian JJ, Janigro D (2010) Extracranial sources of S100B do not affect serum levels. PLoS One 5(9): e12691. doi:10.1371/journal.pone.0012691 

